# Nuclear localization of the caspase-3-cleaved form of p73 in anoikis

**DOI:** 10.18632/oncotarget.6329

**Published:** 2015-10-26

**Authors:** Samar Alsafadi, Sophie Tourpin, Nadia Bessoltane, Sophie Salomé-Desnoulez, Gilles Vassal, Fabrice André, Jean-Charles Ahomadegbe

**Affiliations:** ^1^ Gustave Roussy, INSERM U981, Univ Paris-Sud, F 94805 Villejuif, France; ^2^ Department of Biopathology, Gustave Roussy, F 94805 Villejuif, France; ^3^ Imaging and Cytometry Platform, Gustave Roussy, F 94805 Villejuif, France; ^4^ IRCIV, Univ Paris-Sud, F 94805 Villejuif, France; ^5^ Faculté de Pharmacie, Université de Picardie Jules Vernes, 80000 Amiens, France

**Keywords:** cleaved p73, anoikis, nuclear localization

## Abstract

The transcription factor p73 is a homologue of p53 that can be expressed as pro- or anti-apoptotic isoforms. Unlike p53, p73 is rarely mutated or lost in cancers and it is found to replace defective p53 inducing apoptosis. Here, we investigated the p73 involvement in anoikis, a type of apoptosis caused by inadequate cell-matrix interactions. Breast cancer cell lines with different p53 status were treated with doxorubicin (DOX) or docetaxel (DOC) and cells detached from the extracellular matrix were analyzed. We demonstrate for the first time that DOX-induced cell detachment is associated with p73 cleavage and caspase activation, independently of the p53 status. However, we did not detect p73 cleavage or caspase activation in detached cells under DOC treatment. Overexpressing the apoptotic isoform of p73 led to cell detachment associated with p73 cleavage and caspase activation. Interestingly, p73 cleaved forms localize to the nucleus during the late phase of cell death indicating an increase in the transcriptional activity. Our study suggests that the cleavage of p73 on specific sites may release its pro-apoptotic function and contribute to cell death.

## INTRODUCTION

Epithelial cell survival requires normal cell-cell and cell-extracellular matrix (ECM) signal interactions. When these signals are interrupted under stress conditions, normal cells undergo programmed cell death through a process called anoikis [1, 2]. Certain cancer cells, however, are able to survive this process and stimulate tissue invasion [3]. Resistance to anoikis is a common feature of many tumor cells that has been associated with tumor progression and metastasis [4, 5]. Referring to a complex process *in vivo* environment, anoikis is frequently represented by cell detachment in cell lines [1–4].

The tumor suppressor protein p53 has been described as implicated in anoikis. Indeed, the absence of appropriate signals from the ECM can trigger a p53-regulated apoptosis pathway. Moreover, detached cells bearing p53 mutations have been found to be no longer anchorage-dependent for growth and survival [6, 7]. Unlike p53, the p53 homologue p73 is rarely mutated or lost in cancers. Previous work by our team showed that p73 is capable of replacing defective p53 and inducing apoptosis in response to certain chemotherapeutic agents [8, 9]. Other studies, in turn, showed that p73 is required for efficient cellular response to chemotherapy and DNA damage in cancer cells [10–13]. Although p73 has been described as implicated in response to genotoxic stress, its direct functions are still a subject of debate [14–16]. p73 encodes two classes of isoforms described as having opposing functions: 1) a full-length transactivation-competent p73 protein (TAp73) with tumor suppressor activity; and 2) a group of N-terminally truncated, transactivation-deficient p73 proteins (collectively named ΔTAp73) with oncogenic activity [14, 17–19]. However, a number of studies challenged this assumption. The oncogenic activity of truncated isoforms (ΔTAp73) was disproved [20, 21], and a truncated ΔNp73 isoform was even shown to suppress cell growth under certain conditions [22, 23]. Furthermore, the anti-apoptotic full-length isoform (TAp73β) was shown to promote cell survival by inducing pro-survival caspase 2s, and to counteract cellular senescence [24, 25]. TAp73α was also shown to promote cell survival upon low levels of DNA damage [26]. The opposing functions of each p73 isoform would thus suggest post-transcriptional modifications. Mechanisms that regulate the activity of p73 have not been fully clarified. Given its ability to function as a transcription factor, the activity of p73 may be tightly correlated with its nuclear localization. To be noticed, p53 and p73 contain nuclear localization signals (NLSs) and nuclear export signals (NESs). Mutation or deletion of NLSs leads to cytoplasmic sequestration and a consequent decrease in the transcriptional activity [27, 28]. Interestingly, p73 mutant proteins lacking either NLS or NES were more stable compared with wild-type p73, suggesting that both nuclear import and nuclear export are required for efficient p73 degradation [28].

In our study, we induced anoikis by p73 and p53 gene overexpression or by treatment with doxorubicin (DOX) and docetaxel (DOC), two chemotherapeutic agents widely used in the treatment of breast cancer. We provide clear evidence underpinning the involvement of cleaved forms of p73 in DOX-induced anoikis. Our findings suggest that p73 is cleaved by caspase 3 during anoikis induced by DOX treatment and p73 overexpression. Very interestingly, cleaved forms of p73 localize to the nucleus during the late phase of cell death, indicating a possible increase in p73 transcriptional activity. To our knowledge, our study is the first to describe the nuclear localization of cleaved p73 during the late phase of cell death, suggesting that cleaved p73 forms contribute to cell death.

## RESULTS

### Doxorubicin and docetaxel-induced cell death is mainly preceded by cell cycle arrest and cell detachment

Six breast cancer cell lines with different p53 status were used in our study. Both doxorubicin (DOX) and docetaxel (DOC) treatment induced the detachment of cells from the extracellular matrix (ECM). Drug-treated attached cells and most detached cells excluded trypan blue, i.e. viable cells. Approximately 40% of detached cells readhered and survived when reseeded with fresh drug-free medium.

**Figure 1:**
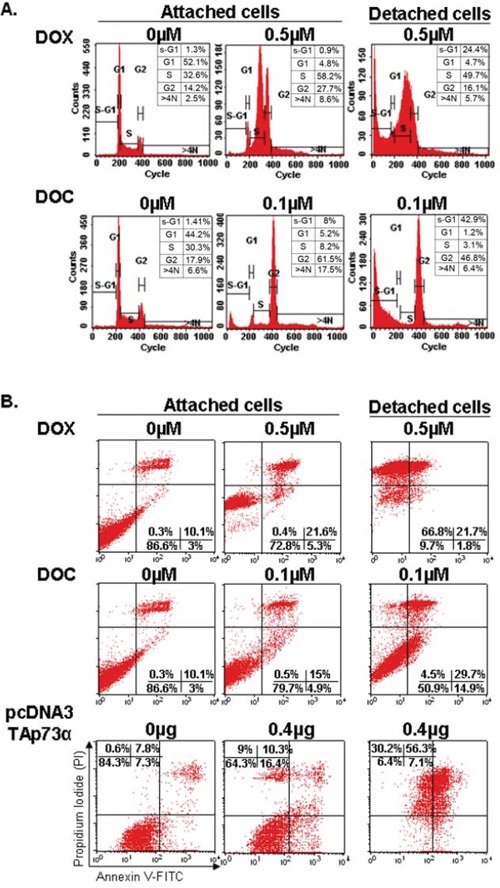
Cell cycle analyses and apoptosis detection in the ZR75–1 cell line by flow cytometry **A.** Cell cycle analysis. FACS analysis was employed to monitor changes in the DNA of cells treated with doxorubicin (DOX) or docetaxel (DOC) as described in the experimental procedure. DOX treatment blocked the cell cycle in phase S while an obvious G2/M phase arrest was observed with DOC treatment. Apoptosis (sub-G1) was only detected in detached cells under treatment (anoikis). The numbers indicate the percentage of cells in each phase of the cell cycle. *Note that the scale of counts is not the same for each panel*. **B.** Apoptosis detection. Apoptosis was quantitated using Annexin V-FITC/PI staining followed by flow cytometry analyses in ZR75–1 cell lines treated with DOX or DOC or transfected with TAp73α. The numbers show the percentages of cells in each quadrant (lower left: FITC−/PI−, intact cells; lower right: FITC+/PI−, early apoptotic cells; upper left: FITC−/PI+, necrotic cells; upper right: FITC+/PI+, late apoptotic or necrotic cells). An increase in the percentage of attached cells exhibiting positivity for Annexin V/FITC staining can be observed after treatment with both drugs (upper and lower right quadrants). A dramatic increase in late apoptosis and necrotic cells exhibiting positivity for PI (upper left and right quadrants) was detected in detached cells treated with DOX. In contrast, detached cells treated with DOC contained both viable and apoptotic cells. Detached cells following TAp73 overexpression (by transfection) largely exhibited a similar profile to that of detached cells treated with DOX.

Functional p53 (ZR75–1) and p53-deficient (MDA-MB361) breast cancer cell lines were treated with the median IC50 of DOX or DOC. Following 48 hrs of DOX treatment, the percentage of attached cells in S-phase increased dramatically from 32.6% to 58.2%, with a concomitant decrease in the G1-phase population from 52.1% to 4.8%. Interestingly, there was no increase in the sub-G1 population, indicative of apoptosis, in attached cells. In contrast, detached cells presented an increase from 1.3% to 24.43% of the sub-G1 population. At 48 hrs of DOC treatment, the percentage of attached cells in G2-phase increased from 17.9% to 61.5%, while G1-phase population decreased from 44.2% to 5.2%. Apoptosis rate (sub-G1) increased from 1.4% to 42.9% in detached cells (Figure 1A). Taken together, these results suggested that apoptosis following cell cycle arrest in S-phase (DOX) or G2/M-phase (DOC) was essentially detected in treated cells after detachment from the ECM.

To confirm these results, we monitored apoptosis using the Annexin V-FITC/PI staining. Figure 1B shows a slight increase in the percentage of apoptotic cells in attached cells after treatment with both drugs (top and bottom right quadrants). A dramatic increase in late apoptotic and/or necrotic cells, 88.5% compared to 10.4% (top left and right quadrants), was detected in detached cells treated with DOX. The results were similar for ZR75–1 and MDA-MB361 cancer cells. Remarkably, the rate of early apoptosis in detached cells was higher under DOC than under DOX treatment (14.9% vs 1.8%, respectively). In contrast, the rate of late apoptosis or necrosis was higher under DOX treatment, 88.5% compared to 34.2% under DOC treatment (Figure 1A and 1B).

### Unlike docetaxel, doxorubicin induces p53 and p73 cleavage

**Figure 2:**
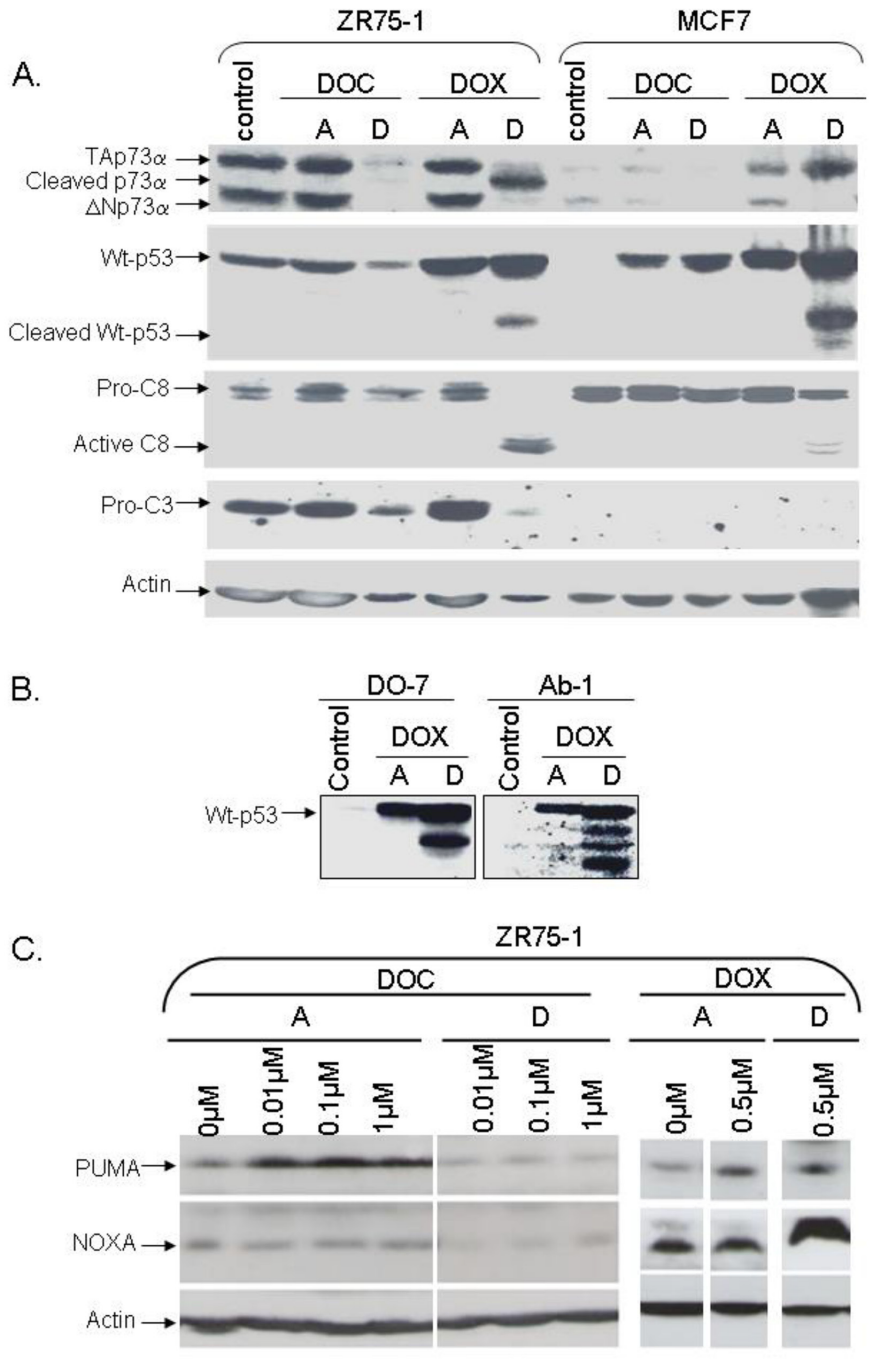
Analysis of protein levels in two wild-type p53 cell lines, ZR75–1 and caspase-3-null MCF-7, following doxorubicin or docetaxel treatment The cells were treated with doxorubicin (DOX) or docetaxel (DOC) with drug concentrations inducing similar toxicity. Following 48 hrs of treatment, attached cells (A) and detached cells (D) were harvested separately for analysis. **A.** Analysis of p53, p73, and caspase-3 and -8 proteins. TAp73α, p53, and caspase 3 were cleaved in detached ZR75–1 cells under DOX treatment. Interestingly, p53, but not p73, was cleaved in caspase-3-null MCF-7. No p53 or p73 cleavage was observed with DOC treatment in either cell line. **B.** Analysis of p53 using DO-7 or Ab-1 antibodies. When p53 was cleaved following DOX treatment, four p53 molecular forms were detected using the Ab-1 monoclonal antibody (directed against residues 376–378) including two forms also recognized by the DO-7 monoclonal antibody (directed against residues 1- 45). **C.** Analysis of Puma and NOXA. Following DOX treatment, Puma levels remained unchanged in the detached cells while NOXA was increased. Curiously, Puma and NOXA expression levels were slightly reduced in detached cells following DOC treatment.

**Figure 3:**
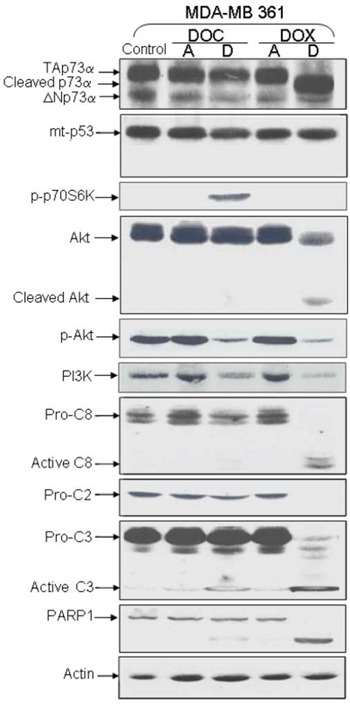
Analysis of protein expression in deficient-p53 MDA-MB 361 following DOX or DOC treatment TAp73α, Akt, Caspases -2, -3, -8, and PARP-1 were cleaved in detached cells treated with DOX. Mutated p53 was not cleaved. Neither p53 nor p73 cleavage was observed with DOC treatment. The level of phosphorylated Akt (pAkt) was decreased in both detached cells after treatment while the p-p70S6K level was increased in DOC-detached cells.

p53 and p73 proteins were analyzed in cell lines treated with DOX or DOC. Surprisingly, a shorter form of TAp73, replacing the TAp73 isoform, was detected in the detached cells under DOX treatment (Figures 2 and 3). The emergence of this new form was independent of p53 status as it was observed in ZR75–1 (wild-type p53) as well as in MDA-MB468, MDA-MB361, and MDA-MB157 (mutated or non-functional p53) cell lines (Figures 2 and 3, data not shown). The new form, however, was not present in MCF-7 (caspase-3-null) detached cells under DOX treatment. To be noticed, DOC treatment did not induce the new form in any of the cell lines.

Using an antibody directed against aa 1–45 (DO-7), we also detected a shorter p53 form in both attached and detached cells treated with DOX. This new p53 form was detected in cell lines with wild-type p53 (MCF-7 and ZR75–1). Interestingly, four p53 molecular forms were detected using an antibody directed against other p53 residues (aa 376–378, Ab-1 monoclonal antibody), including the two forms also recognized by the DO-7 monoclonal antibody (Figure 2). Of note, the new p53 forms were not detected in any of the mutated p53 cell lines under treatment (Figure 3).

### Caspase 3 induces p73 cleavage

To investigate a possible link between cleavage of p53 family members and caspase activity, we analyzed caspase levels in ZR75–1 and MDA-MB361 cells. Caspases 2, 3, 8 were noticeably cleaved in detached ZR75–1 and MDA-MB361 cells following DOX treatment (Figures 2 and 3). PARP-1, a substrate for active caspase 3, was also clearly cleaved. As shown in Figure 2A and as previously described [29], MCF-7 is a caspase-3-null breast cancer cell line. Interestingly, unlike p53 cleavage, PARP-1 and p73 cleavage was not detected in detached MCF-7 cells under DOX treatment.

To confirm the role of caspases in the cleavage of p53 and p73, we treated cells with the cell-permeable, broad-spectrum tetrapeptide caspase inhibitor (zVAD-fmk) 1 hr before adding the drugs. The toxicity of both drugs was largely reduced. A slight cleavage of p53 in attached cells treated with DOX was observed but no cell detachment was detected. We were therefore unable to check the cleavage of p73 in detached cells after caspase inhibition and DOX treatment. Curiously, cell detachment was detected following DOC treatment and caspase inhibition.

NOXA and Puma, two p53-inducible proapoptotic members of the Bcl-2 family, were also analyzed. DOX induced NOXA in detached cells and had no effect on PUMA levels (Figure 2C). Curiously, both NOXA and Puma levels were reduced following DOC treatment in detached cells (Figure 2C).

Bachelder and colleagues established a link between anoikis and AKT/PKB-mediated survival by demonstrating that AKT/PKB was cleaved by caspases in matrix-detached epithelial cells [30]. It has also been demonstrated that the phosphorylation of the p70S6K protein induces P13/AKT pathway deactivation [31]. We found that p-AKT (phosphorylated active form of AKT) levels were decreased in detached cells following treatment. This decrease can be explained by AKT cleavage in DOX-treated cells, and by p70S6K phosphorylation in DOC-treated cells (Figure 3).

### p73 and p53 transfection induces anoikis

Overexpression of TAp73 and, more surprisingly, of ΔNp73 has been shown to stimulate toxicity in cell lines [23]. We thus investigated the effect of the overexpression of TA and ΔNp73 isoforms on cellular death.

**Figure 4:**
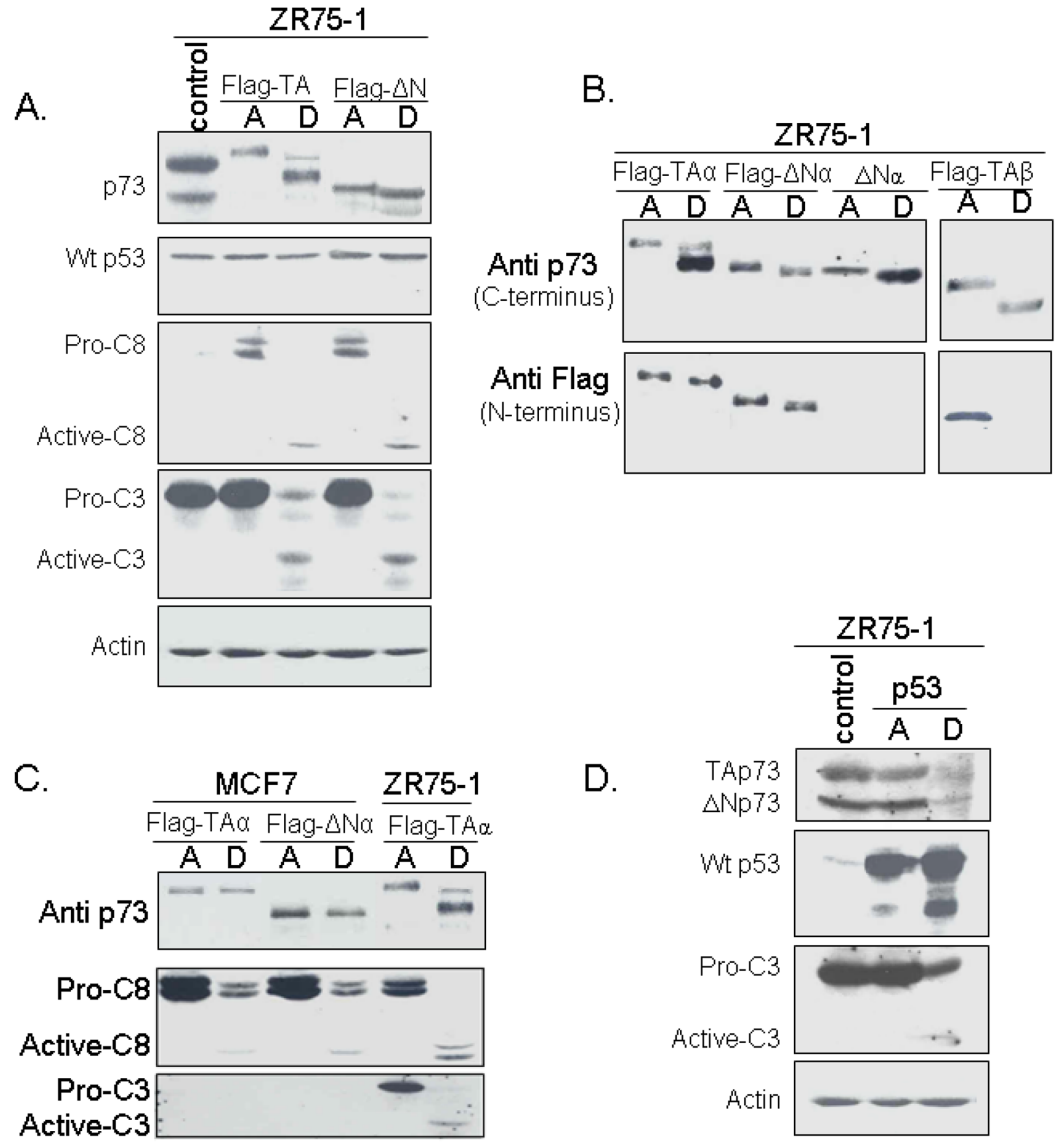
Analysis of protein levels and cleavage following p73 and p53 transfection ZR75–1 was transfected with plasmids bearing different p73 isoforms or p53. Attached (A) and detached (D) cells were harvested separately and analyzed by Western blotting. **A.** Transfection by Flag-TAp73α or Flag-ΔNp73α. The cleavage of TAp73α or ΔNp73α was observed in detached ZR75–1 cells following transfection but there was no evidence of p53 cleavage. Interestingly, cleavage of caspases -8 and -3 was also observed. **B.** Analysis of p73 using antibodies recognizing either the C- or N-terminus. Two antibodies directed against the C-terminus were used: Ab-4 (for TAp73α or ΔNp73α) and an antibody, generously donated by Dr. Caput (France) for TAp73β, another apoptotic TAp73 isoform. The anti-Flag antibody recognized the N-terminus. **C.** Comparison of p73 cleavage in ZR75–1 and caspase-3-nul MCF-7 following TA or ΔN transfection. Neither TAp73 nor ΔNp73 was cleaved in MCF-7 after transfection. **D.** Transfection of the ZR75–1 cell line with p53. Neither TAp73 nor ΔNp73 was cleaved in the ZR75–1 cell line after transfection with p53. By contrast, p53 and, very interestingly, caspase 3 were cleaved in these cells.

The ZR75–1 cell line was transfected with pcDNA3-based expression plasmids bearing TAp73α, TAp73β, or ΔNp73α generated with an N-terminal Flag-tag, and with non-Flag-tagged ΔNp73α. Surprisingly, TAp73α, TAp73β, and ΔNp73α were found cleaved in detached cells (Figure 4). Indeed, the anti-p73 antibody directed against the p73 C-terminal (ab4) detected two cleaved forms of TAp73α, and one cleaved form of TAp73β or ΔNp73α. Only one of p73 cleaved forms was recognized by the anti-flag antibody (N-terminal) (Figure 4B). Caspases 3 and 8 were also cleaved in detached cells. Importantly, wild-type p53 was neither cleaved nor induced in p73-transfected cells (Figure 4).

In order to confirm the involvement of caspase 3 in p73 cleavage, we transfected the caspase-3-null cell line MCF-7 with Flag-TAp73α or Flag-ΔNp73α. No cleavage of p73 was observed in detached cells, confirming that p73 cleavage is caspase-3 dependent (Figure 4C).

Apoptosis was then monitored in the ZR75–1 cells overexpressing Flag-TAp73α (Figure 1B). Attached cells exhibited an increase in early apoptotic cells, as well as late apoptotic and necrotic cells (upper right and left quadrants). Detached cells, however, exhibited a late apoptosis rate of about 86.45%, similar to that of detached cells treated with DOX (Figure 1B).

Wild-type p53 overexpression in ZR75–1 cells was associated with p53 cleavage in attached and detached cells, although to a greater extent in the latter (Figure 4D). Caspase 3 cleavage was exclusively observed in detached cells. p73 protein levels remained unchanged in p53-overexpressing attached cells and decreased to undetectable levels in detached cells.

### p73 cleaved form localizes to nucleus during the late phase of cell death

**Figure 5:**
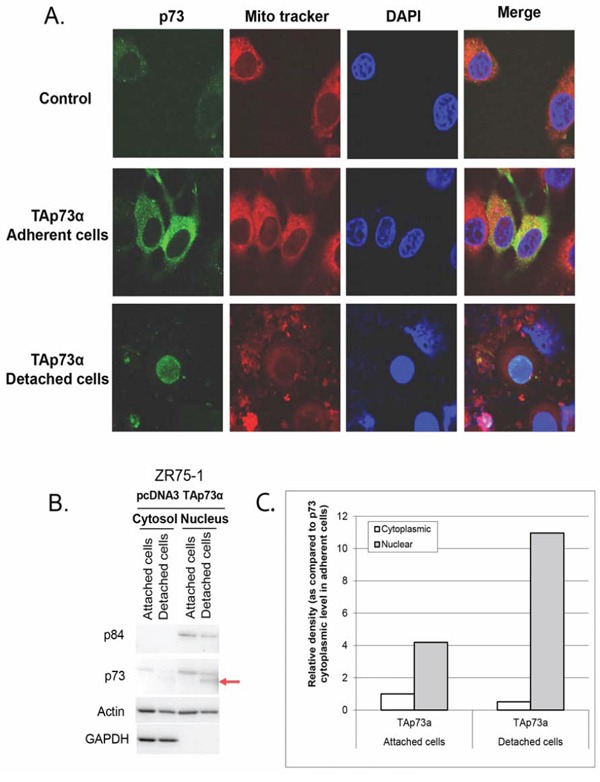
Subcellular localization of p73 forms in ZR75–1 cells **A.** Confocal fluorescence microscopy. ZR75–1 cells were seeded onto glass coverslips overnight and then transfected with TAp73α for 48 hrs. Detached cells were harvested and fixed onto slides. Mitochondria were stained with Mito Tracker red. P73 was stained by anti-p73 (Ab-4), followed by Alexa Fluor 488-conjugated secondary antibody (green). The DAPI counterstained nucleus (Blue). The fluorescence in the overlay (merge) indicates that TAp73α is mostly localized to the cytoplasm in attached cells unlike the cleaved TAp73α form which is localized to the nucleus in detached cells. **B.** Cell fractionation. ZR75–1 cells were transfected with TAp73α. After 48 hrs, detached and attached cells were harvested separately. Cells were resuspended in fractionation buffer and nuclei were separated from the cytoplasm. Equal amounts of proteins were analyzed. Nuclear Matrix Protein p84 and GAPDH were used to check the purity of nuclei and cytoplasm fractions respectively. The level of the TAp73α cleaved form increased spectacularly in the nuclei of detached cells (arrow). **C.** Determination of the levels of TAp73α forms. This analysis confirms a significant decrease in the level of TAp73α in the cytoplasm of the detached cells whereas the rate of the protein increased sharply in the nuclei of these cells with a very predominant expression of the TAp73α cleaved form.

To evaluate whether the cleaved form of p73 possesses a biological function, we investigated its cellular localization. ZR75–1 cells were transfected with TAp73α and we then analyzed separately attached and detached cells. TAp73α was mostly localized to the cytoplasm in attached cells while, surprisingly, its cleaved form localized to the nucleus (Figure 5A).

We proceeded to confirm these findings with a cell fractionation test to isolate the nucleus from whole cell lysates. Nuclear matrix protein p84 and GAPDH were used to check the purity of nuclei and cytoplasmic fractions respectively. In attached cells where the cleaved form was undetectable, TAp73α was more abundant in the nucleus than in the cytoplasm. Interestingly, TAp73α cleaved form was exclusively present in the nuclei of detached cells (Figure 5B and 5C). This nuclear localization implies a transcriptional activity of cleaved p73.

## DISCUSSION

This study demonstrates for the first time, that the cleavage of TAp73α is involved in anoikis induced by both DOX treatment and TAp73α overexpression. Importantly, DOX induced p53 cleavage during anoikis, but only in wild-type p53 cell lines. Of note, neither p53 nor TAp73α cleavage was triggered in DOC-induced anoikis. Very interestingly, cleaved forms of TAp73α localize to the nucleus during the late phase of cell death, indicating a transcriptional activity of the cleaved form.

**Figure 6:**
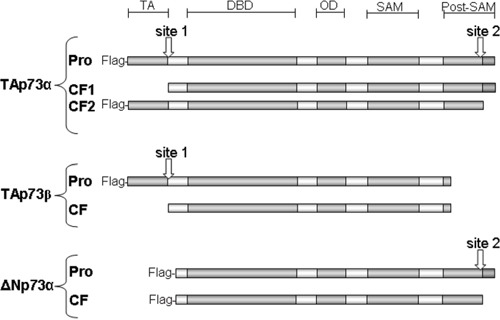
Schematic diagram of TAp73α, TAp73β and ΔNp73α, and cleavage sites Different antibodies recognizing either the N-terminus (Flag antibodies) or the C-terminus of p73 forms were used to estimate the location of cleavage sites. TAp73α contains two possible sites that can generate two different cleaved forms which are detected in detached cells after TAp73α transfection. On the other hand, only one cleaved form was generated by the cleavage of TAp73β and ΔNp73α. TAp73β probably contains the N-terminal site, while ΔNp73α contains only the C-terminal site. Merging these results with the sequences of p73 forms, the N-terminal cleavage site is possibly located in one of five aspartate residues at that end while the C-terminal cleavage site is located in one of seven aspartate residues. TA, transactivation domain; DBD, DNA-binding domain; OD, oligomerization domain; SAM, Sterile alpha motif domain; Post-SAM, post sterile alpha motif domain. Pro, proform before cleavage; CF, cleaved form.

Anoikis is a type of cell death induced by inadequate cell-matrix interactions [1–4]. Very few studies have analyzed cleavage in p53 family members and its possible role in anoikis. One study reported that cleaved p53 at the N-terminus is likely to be competent for the transactivation of p53 target genes and the induction of apoptosis [32]. The cleavage product loses a major part of the mdm2 binding interface and can thus be resistant to feedback regulation by mdm2, leading to p53 accumulation [33]. Furthermore, wild-type p53 has been described as a latent form of p53 that does not bind to DNA. This latent state of p53 depends on a C-terminal negative regulatory domain that locks the unphosphorylated p53 tetramer in an inactive state [34]. Accordingly, the p53 C-terminally truncated isoforms induce the expression of IGFBP3, whereas the full-length ones do not [35]. In our study, DOX-induced anoikis was associated with both p73 and wild-type p53 cleavage. Overexpressing different p73 isoforms also induced anoikis and p73 cleavage. We suggest two possible sites in TAp73α that generate two different cleaved forms (Figure 6). When cells were transfected with TAp73β or ΔNp73α, only one cleaved form was generated (Figures 4 and 6). TAp73β probably contains the N-terminal site, while ΔNp73α only contains the C-terminal site. Cleavage on the N-terminal rather than the C-terminal site would be important for an efficient apoptotic response because it is associated with stabilization of cleaved proteins and high toxicity.

One study showed that p73 could be cleaved by caspases 3 and 8 both *in vitro* and *in vivo*, especially during apoptosis elicited by tumor necrosis factor-related apoptosis-inducing ligand (TRAIL) receptor ligation. The authors reported that TAp73 and some of its cleavage products were localized to mitochondria, suggesting a basis for transcription-independent p73-induced death, as previously described for p53 [36–38]. Of note, we found that p73 cleavage occurred later during anoikis. Based on our findings and those of these authors, p73 cleaved forms might translocate to mitochondria and induce apoptosis in a transactivation-independent way during anoikis. Unfortunately, the fragility of detached cells during anoikis complicated certain analyses such as mitochondria purification and RNAi inhibition.

It has also been reported that cleavage of wild-type and mutated p53 generates four fragments, two of which translocate to mitochondria and induce mitochondrial membrane depolarization in the absence of transcriptional activity [39]. Accordingly, cleaved mutant p53 induces mitochondrial depolarization, attributing a wild-type p53 function to mutant p53 [39]. Conflicting data, however, have been reported in other studies in which tumor-derived p53 mutants were incapable of or severely impaired in releasing cytochrome c [36, 37]. In our study there was no cleavage of mutated p53 in treatment-induced anoikis, possibly leading to impaired mitochondrial permeabilization (Figure 3). Consequently, missense mutations in tumors might select against both transcriptional and mitochondrial apoptotic activity of p53.

p53 was cleaved in the null-caspase-3 breast cancer cell line (MCF-7), while p73 cleavage was totally inhibited. This finding shows for the first time that p73 cleavage, unlike p53 cleavage, is dependent on caspase-3 activation. We used a caspase inhibitor to demonstrate the role of caspase 3 in p53 and p73 cleavage during anoikis. Nevertheless, this agent decreased drug activity, leading to the absence of cell detachment and blockage of anoikis.

TAp73α was significantly cleaved in detached cells following TAp73α overexpression. Wild-type p53 was neither induced nor cleaved in these cells. Inversely, TAp73α was not cleaved in anoikis induced by p53 overexpression. Taken together, our results imply that TAp73α cleavage and p53 cleavage use two independent pathways. This could be a protection mechanism: if one of the pathways is disrupted, cells would still be able to use the second one.

When anoikis was stimulated through cell transfection with TAp73α or TAp73β plasmids, detached cells exhibited cleavage of TAp73 and an Annexin/IP profile that were comparable to that of DOX-induced anoikis (Figures 1 and 4). We therefore transfected ZR75–1 cells with TAp73α, and analyzed the subcellular localization of normal and cleaved forms of p73. The results showed that TAp73α was localized in the cytoplasm of attached cells. This cytoplasmic protein is incapable of exerting transcriptional activities. In contrast, the very high concentration of TAp73α in the nuclei of detached cells, suggests strong transcriptional activity of TAp73α in these cells. The nuclear localization of TAp73α is associated with caspase activation and strong induction of NOXA (Figures 1B, 2C and 3) [40–42]. Saha and colleagues showed that the treatment of multiple myeloma cells with PRIMA-1Met resulted in activation of caspase-3 and p73, followed by NOXA upregulation. Knockdown of p73 diminished NOXA expression, while silencing NOXA did not modify p73 expression, indicating that NOXA is the downstream target of p73. Functionally, knockdown of either p73 or NOXA resulted in attenuation of PRIMA-1Met-induced apoptosis [40].

Fractionation experiments of nuclei and cytoplasm clearly showed that the TAp73 was present in the cytoplasm of attached cells, and in the nuclei of both attached and detached cells. In contrast, the cleaved TAp73α form appeared only in the nuclei of detached cells. Consequently, induction of apoptosis and proteins involved in apoptosis is associated with the nuclear localization of the cleaved TAp73α. Other authors have shown that, a nuclear import-deficient form of p73 was less capable of activating gene expression in transfected cells, which is consistent with our hypothesis that nuclear localization of p73 is synonymous with transcriptional activity inducing apoptosis [28].

Al-Bahlani and colleagues showed that calpain is required for cisplatin-induced apoptosis in ovarian cells [43]. Calpain co-localized with TAp73α and was able to cleave TAp73α at both the N- and the C-terminus. It is not known whether calpain-induced cleavage would result in TAp73α degradation, as in the case of Xiap [44] or enhanced Bax function [45]. The occurrence of calpain-induced TAp73α cleavage in chemosensitive, and not in the chemoresistant cells is consistent with the concept that TAp73α cleavage and activity is involved in the regulation of chemosensitivity. However, the biological relevance of cleaved TAp73α remained unclear. The authors concluded that TAp73α could contribute to the response to cisplatin by translocation into the nucleus [43]. Our work allowed us to clearly show that cleaved TAp73 is translocated into the nuclei of cells, and that this translocation is associated with the induction of apoptosis.

Recently, it has been shown that histone deacetylation inhibition and p73 expression synergistically enhanced the cytotoxicity. This synergy was associated with a decrease of p73 protein level [46]. Such findings indicate that histone acetylation may potentiate the apoptotic function of p73 despite a decrease in p73 protein levels. Likewise, extensive DNA damage was shown to induce apoptosis associated with reduced p73 levels. p73 cleavage by caspases was excluded as no p73 cleaved forms were detected [26]. Yet, detached cells were not considered in these studies. Our findings support that normal p73 reduction is probably du to cleavage releasing its pro-apoptotic function in response to DNA damage, as shown with DOX treatment.

In conclusion, this work is the first to demonstrate the induction and nuclear localization of the cleaved forms of TAp73α during anoikis. p53 and p73 feature a number of cryptic functions that can be selectively activated by specific truncations of the proteins. Based on our findings, we present a model for the activation of p73 by cleavage in anoikis induced by DOX treatment or p73 overexpression. This model could radically improve our understanding of p53 network interactions. However, another mechanism is probably employed by DOC at this stage. Further functional characterization of p73 cleaved forms may open up new possibilities of therapeutic combinations of anti-cancer molecules and gene therapy.

## MATERIALS AND METHODS

### Cell culture and transfections

Human breast cancer cell lines with functional p53 (MCF-7, ZR75–1) or mutated p53 (T47D, MDA-MB157, MDA-MB361, MDA-MB468) [9, 47] were cultured as monolayers at 37°C with 5% CO2, and maintained by regular passage in Dulbecco's modified Eagle medium with Glutamax-1 (Gibco-BRL, Grand Island, NY, USA) supplemented with 10% heat-inactivated fetal bovine serum, 5 μg/mL of fungizone, 12.5 μg/mL of vancomycin, and 10 μg/mL of gentamicin. Twenty-four hours after plating, the cells were treated for 24 hrs and 48 hrs with DOX (Adriblastine^®^, Pharmacia) or DOC (Taxotere^®^, Sanofi-Aventis). For caspase-inhibition experiments, 50 μM of the caspase inhibitor Z-VAD (OMe)-FMK (Calbiochem) were freshly added to the growth medium 1 hr prior to treatment.

After 24 hrs or 48 hrs of treatment, detached and attached cells were harvested separately, counted using the trypan blue exclusion method, and processed for the various biochemical assays. For other experiments, detached cells were sedimented, resuspended in fresh medium, and seeded onto new culture plates.

Transfections were carried out using Lipofectamine 2000 (Invitrogen). Flag-TAp73α, Flag-TAp73β, Flag-ΔNp73α cloned in pcDNA3, ΔNp73α cloned in MSCV-IRES-GFP, and wild-type p53 cloned in a pCMV-Neo-Bam were generous gifts from Alex I. Zaika (Nashville, USA). Cells (4 × 10^5^) were plated out in 6-well plates and transfected 24 hrs later.

### Immunoblotting

Cells were lysed in RIPA buffer and proteins were quantified using a microBCA assay (Pierce, ThermoScientific). Equal amounts were separated on SDS-PAGE gels. Proteins were transferred to PVDF membranes followed by immunoblotting with antibodies specific for: mouse anti-p53 (DO-7, DAKO), mouse anti-p53 (AB-1), mouse anti-p21 (Ab-1), mouse anti-Noxa and rabbit anti-p73 (Ab-4) all from Calbiochem, rabbit anti-p73α and β (generously donated by Dr. Caput, Sanofi Labege, France), mouse anti-flag (M2, Sigma-Aldrich), mouse anti-p84 (ab487, abcam), mouse anti-GAPDH (MAB374, Millipore), goat anti-Puma (N19, Santa Cruz Biotechnology), mouse anti-caspase 8/FLICE (5F7, MBL), rabbit anti-caspase 3, mouse anti-caspase 2, rabbit anti-Akt, rabbit anti-pSer473-Akt, and mouse anti-PARP-1 (Ab-2), all from Cell Signaling Technology. Proteins were visualized using an enhanced chemiluminescence detection system (Amersham). Membranes were then washed in 0.5% TBS-Tween, and incubated with 0.2 μg/mL of mouse anti-actin (C4, Chemicon) to quantify and normalize results.

### Immunofluorescence and confocal microscopy

ZR75–1 cells were seeded onto glass coverslips in 6-well plates overnight and transfected with TAp73α for 48 hrs. Detached cells were harvested and fixed onto slides by using cytospin (Shandon, Inc). For mitochondria staining, 100nM mitotracker (M22425, Molecular Probes Inc) were added to the culture medium for 15 minutes before fixing with 4% paraformaldehyde in PBS for 30 minutes at room temperature (RT). Fixed cells were washed with PBS and permeabilized with 0.05% Tween-20 in PBS for 10 minutes. After washing with PBS, the coverslips were incubated with anti-p73 (Ab-4, Calbiochem), diluted in 5% BSA/PBS for 1 hr at RT. They were then washed twice with PBS, followed by incubation with Alexa Flour 488 conjugated anti-rabbit IgG (Molecular Probes Inc) for 30 minutes at RT. After washing 3X with PBS, the coverslips were mounted with Vectashield^®^ containing DAPI (Vector Labs, CA). Cells were examined under a Leica SpE confocal microscope.

### Cell fractionation

ZR75–1 cells were transfected with TAp73α for 48 hrs. Detached and attached cells were harvested separately. ZR75–1 cells were washed with cold PBS, followed by centrifugation at 12000g for 5 minutes. The cell pellet was resuspended in fractionation buffer (10 mM HEPES pH 7.9, 1.5 mM MgCl2, 10 mM KCl, 0.5 mM dithiothreitol) and protease inhibitors (cOmplete Mini EDTA-free Protease Inhibitor Cocktail Tablets, Roche). After 10 minutes incubation on ice, 10% NP40 were added followed by centrifugation. The supernatant was removed and used as a cytoplasmic fraction. The pellet was washed once and lysed with lysis buffer (20 mM HEPES pH7.9, 25% glycerol, 1.5 mM MgCl2, 0.2 mM EDTA, 0.46M NaCl and protease inhibitors) for 15 minutes, spun at 12000g for 5 minutes and the supernatant was used as a nuclear extract. Proteins were quantified using a microBCA assay (Pierce, ThermoScientific).

### Cell cycle analysis

Cell cycle distribution was measured in untreated cells and cells treated with chemotherapeutic agents. Detached and attached cells were harvested separately 24 and 48 hrs after treatment, suspended in PBS, and fixed in 70% ethanol. DNA content was evaluated after propidium iodide staining. Fluorescence-activated cell-sorting analysis was carried out using a FACScan flow cytometer (Beckton Dickinson, San Diego, CA, USA) and CellQuest software.

### Apoptosis detection

The Annexin V-FITC Apoptosis Detection Kit (BD Biosciences) was used to detect apoptosis by flow cytometry. At 48 hrs of treatment, cells were harvested, washed in PBS, and pelleted by centrifugation. They were resuspended at 105 cells/100 μL in a binding buffer to which 5 μL of Annexin-V and 5 μl of propidium iodide were added and then incubated in the dark for 15 min at room temperature. Then, 400 μL of binding buffer were added and the cells were immediately processed with a FACScan flow cytometer.
